# Structural and protein interaction effects of hypertrophic and dilated cardiomyopathic mutations in alpha-tropomyosin

**DOI:** 10.3389/fphys.2014.00460

**Published:** 2014-12-02

**Authors:** Audrey N. Chang, Norma J. Greenfield, Abhishek Singh, James D. Potter, Jose R. Pinto

**Affiliations:** ^1^Department of Molecular and Cellular Pharmacology, Leonard Miller School of Medicine, University of MiamiMiami, FL, USA; ^2^Department of Neuroscience and Cell Biology, Robert Wood Johnson Medical School, Rutgers UniversityNew Jersey, NJ, USA; ^3^Department of Cardiology, UCSF Medical Center, University of California, San FranciscoSan Francisco, CA, USA; ^4^Department of Biomedical Sciences, Florida State University College of MedicineTallahassee, FL, USA

**Keywords:** tropomyosin structure, cardiomyopathy, circular dichroism, thermal denaturation, actomyosin ATPase, molecular modeling

## Abstract

The potential alterations to structure and associations with thin filament proteins caused by the dilated cardiomyopathy (DCM) associated tropomyosin (Tm) mutants E40K and E54K, and the hypertrophic cardiomyopathy (HCM) associated Tm mutants E62Q and L185R, were investigated. In order to ascertain what the cause of the known functional effects may be, structural and protein-protein interaction studies were conducted utilizing actomyosin ATPase activity measurements and spectroscopy. In actomyosin ATPase measurements, both HCM mutants and the DCM mutant E54K caused increases in Ca^2+^-induced maximal ATPase activities, while E40K caused a decrease. Investigation of Tm's ability to inhibit actomyosin ATPase in the absence of troponin showed that HCM-associated mutant Tms did not inhibit as well as wildtype, whereas the DCM associated mutant E40K inhibited better. E54K did not inhibit the actomyosin ATPase activity at any concentration of Tm tested. Thermal denaturation studies by circular dichroism and molecular modeling of the mutations in Tm showed that in general, the DCM mutants caused localized destabilization of the Tm dimers, while the HCM mutants resulted in increased stability. These findings demonstrate that the structural alterations in Tm observed here may affect the regulatory function of Tm on actin, thereby directly altering the ATPase rates of myosin.

## Introduction

Numerous mutations in the proteins of the sarcomere have been associated with cardiomyopathies (Chang and Potter, 2005; Force et al., 2010; Seidman and Seidman, 2011; Hershberger et al., 2013). Various studies of cardiomyopathic mutations suggest distinct mechanisms for the progression of the dilated cardiomyopathic (DCM) and hypertrophic cardiomyopathic (HCM) phenotypes (Chang et al., 2005; Robinson et al., 2007; Willott et al., 2010). An understanding of the disease progression necessitates the study of the effects of the mutations on protein structure and function (Force et al., 2010; Tardiff, 2011). One of the sarcomeric proteins found to have mutations associated with cardiomyopathies is cardiac α-tropomyosin (Tm) (Redwood and Robinson, 2013). Tm is a semi-flexible rod-like protein whose coiled-coil structure functions to control myosin interaction sites on actin (Perry, 2001). Troponin (Tn) confers Ca^2+^ sensitivity to this function (Holroyde et al., 1980; Farah and Reinach, 1995; Gordon et al., 2000). Putative actin on/off regions along the length of Tm have been known for some time (McLachlan and Stewart, 1976), and more recent studies of these regions have demonstrated that although the length of the Tm dimer is composed of regular turns which correspond to seven actin binding regions, those regions are not all equal in terms of structure and contribution to thin filament function (Phillips et al., 1986; Hammell and Hitchcock Degregori, 1997; Landis et al., 1997, 1999; Singh and Hitchcock-Degregori, 2003; Barua et al., 2013).

How a single point mutation out of 284 amino acids in Tm can lead to cardiomyopathies is an interesting question which this paper attempts to understand, through the study of four mutations, the DCM associated mutations E40K and E54K, and the HCM associated mutations E62Q and L185R. All of these mutations were previously identified from patients who presented severe cardiomyopathy (Olson et al., 2001; Van Driest et al., 2002; Jongbloed et al., 2003). Previous investigation on the effects of these HCM and DCM mutations by myofibrillar ATPase measurements showed an increase in Ca^2+^ sensitivity for HCM and a decrease in Ca^2+^ sensitivity for DCM associated mutations, which is commonly found in mutations of the thin filament associated with these diseases (Chang et al., 2005). In addition, a decrease in inhibition of ATPase activity at low Ca^2+^ concentrations were reported for the HCM associated mutations; while DCM associated mutations had no significant effect on inhibition, suggesting that distinct mechanisms, at the thin filament level, may underlie the different disease phenotypes (Chang et al., 2005). The causes of the distinct functional effects previously reported are unknown, and while semi-“intact” systems such as reconstituted myofibrils provide important mechanistic information, there are too many variables to identify the causative perturbation.

Reconstituted fiber studies have shown distinct changes in actin binding properties and actomyosin interactions linked to altered movement of Tm on actin caused by DCM associated mutations E40K and E54K (Borovikov et al., 2009b,c; Bai et al., 2012). Results of such studies have led to conclusions that distinct mutations cause defects in contraction via mechanisms unique to the mutations (Borovikov et al., 2009a,b,c; Bai et al., 2012). Reconstituted fibers are complex intact systems in which the components of the thin filament are completely replaced with exogenous proteins. More recently, energy landscape studies have shown that mutations in Tm linked to HCM are associated with a decrease in actin–Tm interaction energy, which is thought to impair the relaxation properties of the thin filament (Orzechowski et al., 2014). However, using the same technique, mutations in Tm linked to DCM do not seem to enhance actin-Tm interaction (Orzechowski et al., 2014). Due to the complexity of the system, here we aimed to understand the effects of the mutations on Tm's interactions with its functional partner proteins by actomyosin ATPase activity assays and on the helical stability of Tm dimers. Unlike reconstituted myofibrillar or fiber preparations, actomyosin ATPase activity assays were performed with purified actin, myosin, Tm and Tn. By titrating Tm and Tn into actin and myosin mixtures, we sought to identify the specific protein-protein interaction affected by the abovementioned point mutations in Tm. Results of these studies pointed toward the possibility of effects of the mutations on Tm dimer interactions, which have the potential for long-range effects on myofilament function. The structural effects of the mutations were further dissected through thermal denaturation studies and energy minimization studies.

## Materials and methods

### Expression and purification of recombinant Tms and Tns

Recombinant Tm and Tns were expressed and purified utilizing previously described methods (Chang et al., 2005). Alanine-Serine were added to the N-terminus of all Tm proteins to mimic acetylation as previously described (Urbancikova and Hitchcock-Degregori, 1994), and is referred to as “ASWT” in figures. Human cardiac Tn complexes comprised of recombinant TnT, TnI, and TnC were prepared by sequential dialysis in decreasing concentrations of urea and KCl (Gomes et al., 2002).

### Thermal denaturation studies by circular dichroism (CD)

Far-UV CD spectra were collected using a 1-mm-path quartz cell in a Jasco J-720 spectropolarimeter. The measurements were obtained using pure recombinant proteins which were dialyzed in 10 mM sodium phosphate buffer pH 7.0, 150 mM sodium fluoride, and diluted to 0.1 mg/mL. Thermal denaturation measurements were obtained with proteins which had been diluted to 0.1 mg/mL, at 222 nm over a range of temperatures (20–60°C). Melting temperatures were determined by fitting the mean residue ellipticity, [θ]_222_, to a two-state or three-state transition model as previously described (Greenfield and Hitchcock-Degregori, 1995; Greenfield, 2006). See description for the three-state transition model below:

(1) $$\theta_{observed}=\epsilon_{1}\alpha_{1}+\epsilon_{2}\alpha_{2}+\epsilon_{2}\alpha_{2}$$

where

(2) $$\begin{array}{l} \alpha_{1}=\frac{K_{1}}{1+K_{1}},\alpha_{2}=\frac{K_{2}}{1+K_{2}},\;and\\ \alpha_{3}=\frac{\left(4[C]K_{3}+1-\sqrt{8[C]K_{3}+1}\right)}{4[C]K_{3}} \end{array}$$

and

(3) $$\begin{array}{l} K_{1}=e^{\left(\frac{\Delta H_{1}}{RT}\right)\left(\frac{T}{T_{M1}}-1\right)},K_{2}=e^{\left(\frac{\Delta H_{2}}{RT}\right)\left(\frac{T}{T_{M2}}-1\right)},\\ K_{3}=e^{\left(\frac{\Delta H_{3}}{RT}\right)\left(\frac{T}{T_{M3}}-1\right)-In[C]} \end{array}$$

Δ*H*_1_, Δ*H*_2_, andΔ*H*_3_ are the respective enthalpies of folding of the first, second, and third transitions at the lowest to highest temperature. *T*_*M*1_, *T*_*M*2_, and *T*_*M*3_ are the observed midpoints of each transition. ∈_1_, ∈_2_, ∈_3_ are the extinction coefficients for the circular dichroism change associated with each transition, and [C] is the concentration of the Tm dimer in mole/L. The initial values of ΔH and *T_M_* were estimated and the unfolding equations were fit using SigmaPlot 11.0 (Greenfield, 2006). The ASWT and L185R were fit using a two-state transition model, while the other mutants were fit using a three-state transition model as determined by the correlation coefficient (Greenfield, 2006).

### Purification of rabbit skeletal actin

Skeletal actin was purified from the longissimus dorsi and psoas muscles of female New Zealand White rabbits, utilizing routine methods established in the lab, according to the procedure described by Strzelecka-Golaszewska et al. (1975) and Pardee and Spudich (1982).

### Purification of porcine cardiac myosin

Cardiac myosin was purified from the left ventricles of hearts from freshly sacrificed pigs, by a method routinely utilized in the lab (Szczesna et al., 2000), based on the original procedure described by Murakami et al. (1976).

### Actomyosin ATPase assays

Porcine cardiac myosin, rabbit skeletal actin and human cardiac Tm and Tns were homogenized to a final concentration of 0.6 μM myosin, 3.5 μM actin, 1 μM Tm, and 0–1.5 μM Tn in ATPase assay buffer (40 mM KCl, 15 mM MOPS pH 7.0, 3.5 mM MgCl_2_, 1 mM DTT, 1 mM EGTA (pCa 9.5) or 0.416 mM CaCl_2_ (pCa 4). For Tm titration studies, Tm was varied from 0 to 1.5 μM, in the absence of Tn. ATPase reactions were initiated with the addition of ATP to a final concentration of 2.9 mM, the amount calculated to yield approximately 1 mM free MgCl_2_ (Dweck et al., 2005). ATPase assays were performed at 37°C for 20 min and the reaction was stopped with the addition of 25 μL of 35% trichloroacetic acid. The amount of inorganic phosphate released during the ATPase assays was determined by use of the Fiske-Subbarrow reagent (Fiske and Subbarrow, 1925).

### Statistical analysis

For all measurements, significance of comparison to ASWT was determined by 1- or 2-way ANOVA followed by Dunnett's multiple comparisons test against ASWT. In all figures significance is denoted as follows: ^*^p < 0.05, ^†^*p* < 0.01, ^‡^*p* < 0.001.

### Mutant Tm modeling

To model the effects of the E40K, E54K, and E62Q mutations, the structure of a fragment of Tm containing residues 1–80, pdb accession number 1IC2 were used as a starting point (Brown et al., 2001). To model the effect of the mutation L185R the X-ray structure of a mid-region fragment of Tm, pdb accession number 2B9C was used as a starting point (Brown et al., 2005). In all cases, the point mutations were introduced into the respective crystal structures, hydrogens and Gastiger- Huckel charges were added to the structure, and the structures were then minimized with unrestrained helices to convergence using the program Sybyl (Tripos Associates). The molecular graphics images were produced using the UCSF Chimera package from the Computer Graphics Laboratory, University of California, San Franscisco (Pettersen et al., 2004) supported by National Institutes of Health grant P41 RR-01081.

## Results

### Effects of mutations on Tn concentration dependent activation and inhibition of actomyosin ATPase activity

**Figure 1:**
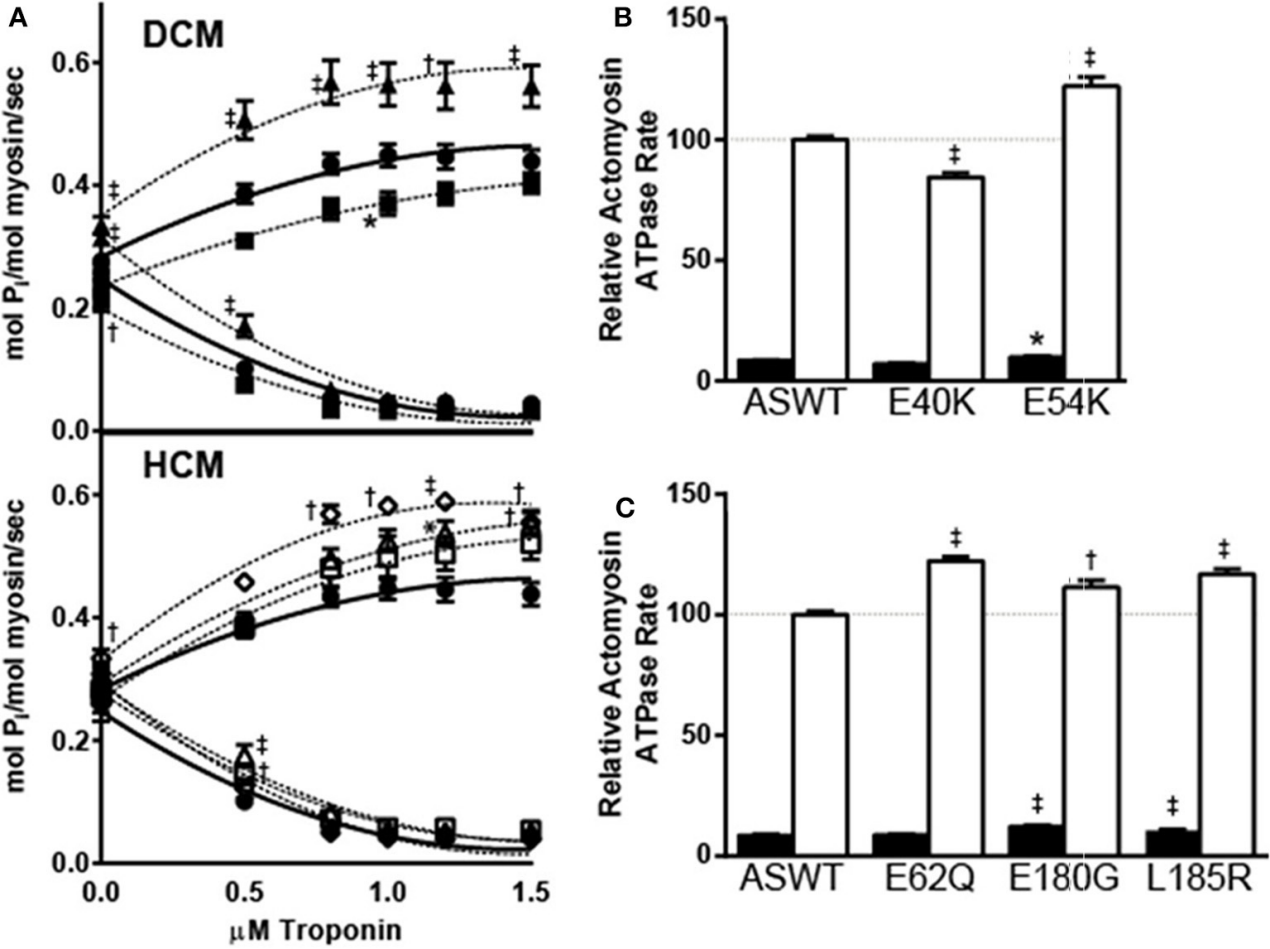
**Effects of DCM and HCM Tm mutants on Tn concentration dependent actomyosin ATPase activity. (A)** The Tn concentration-dependent ATPase rates of actomyosin samples prepared with ASWT and mutant Tms were compared. Tn was titrated from 0 to 1.5 μM into mixtures of 0.6 μM myosin, 3.5 μM actin, and 1 μM Tm, and the actomyosin ATPase activation at high (pCa 4) [Ca^2+^] (up-sloping) and inhibition at low (pCa 9.5) [Ca^2+^] (down-sloping) were measured for DCM (upper panel) and HCM (lower panel) associated mutations. The results represent the mean ± SE of calculated activity rates of experiments (*n* = 5) performed in triplicate. Samples graphed on DCM panel: ASWT (filled circle), E40K (filled square), E54K (filled triangle); on HCM panel: ASWT (filled circle), E62Q (open diamond), E180G (open square), L185R (open triangle). The maximal ATPase activity values of ASWT and DCM-associated **(B)** and HCM-associated **(C)** mutant Tm reconstituted actomyosin solutions in low (pCa 9.5; filled bar) and high (pCa 4; open bar) [Ca^2+^] were compared. Relative rates shown graphed are a percentage of the mean high [Ca^2+^] activated ASWT rate obtained from mixtures of 0.6 μM myosin, 3.5 μM actin, 1 μM Tm, and 1–1.5 μM Tn. Mean percentages ± SE were graphed; *n* = 20. Statistical significance were determined by ANOVA followed by Dunnett's multiple comparisons test; ^*^p < 0.05, ^†^*p* < 0.01, ^‡^*p* < 0.001.

To investigate the effects of the mutations on actin-myosin interaction and thin filament regulation of ATPase activity, the actin and myosin concentrations were held constant and the effects of increasing concentrations of Tn on actomyosin ATPase rates were measured. As a control for the assay, a well-characterized HCM mutation E180G was also included in the actomyosin ATPase assays. The results of these studies show that the two DCM mutants gave strikingly different results where E40K causes a significant decrease in maximal ATPase activity and E54K causes an increase (Figure 1A). Increased rates over ASWT were measured at high [Ca^2+^] for all three HCM mutants (Figure 1A).

In all assays, ATPase rates reached a maximum by 1 μM Tn. Therefore, relative maximal actomyosin ATPase rates of DCM (Figure 1B) and HCM (Figure 1C) mutant Tm samples in high and low [Ca^2+^] at 1 μM Tm and 1–1.5 μM Tn, were plotted to better illustrate the [Ca^2+^]-dependent trends. When normalized to ASWT in high [Ca^2+^], the rate at low [Ca^2+^] is 8.5 ± 0.3%. Consistent with plots in Figure 1A, the maximal rates for E40K in high and low [Ca^2+^] were lower: 85 ± 1.8% and 7.1 ± 0.1%, respectively. The maximal rates for E54K in high and low [Ca^2+^] were higher: 122 ± 3.8% and 9.9 ± 0.4%, respectively. All HCM associated mutants showed significantly increased rates in high [Ca^2+^], of 122 ± 1.7% for E62Q, 112 ± 2.9% for E180G, and 117 ± 2.1% for L185R. In low [Ca^2+^], all but E62Q had significantly increased rates: 8.7 ± 0.3% for E62Q, 12 ± 0.5% for E180G, and 11 ± 0.5% for L185R.

In addition to the differences in maximal Ca^2+^-dependent activity, the rates in the absence of Tn vary significantly for some mutants, suggesting that Tm alone is affecting the actomyosin ATPase rate (Figure 1A). In the absence of Tn, the actomyosin ATPase activity is not Ca^2+^-dependent. Comparison of averaged baseline values in the absence of Tn showed that the ATPase activity in mol Pi/mol myosin/sec was 0.27 ± 0.01 for ASWT, and significantly lower at 0.23 ± 0.1 for E40K. The baseline activity was significantly higher for E54K and E62Q, which were both 0.32 ± 0.1. The baseline activity differences were not statistically significant for E62Q and E180G, which were 0.28 ± 0.02 and 0.30 ± 0.01.

### Effects of the mutations on the Tm concentration dependent inhibition of actomyosin ATPase activity

**Figure 2:**
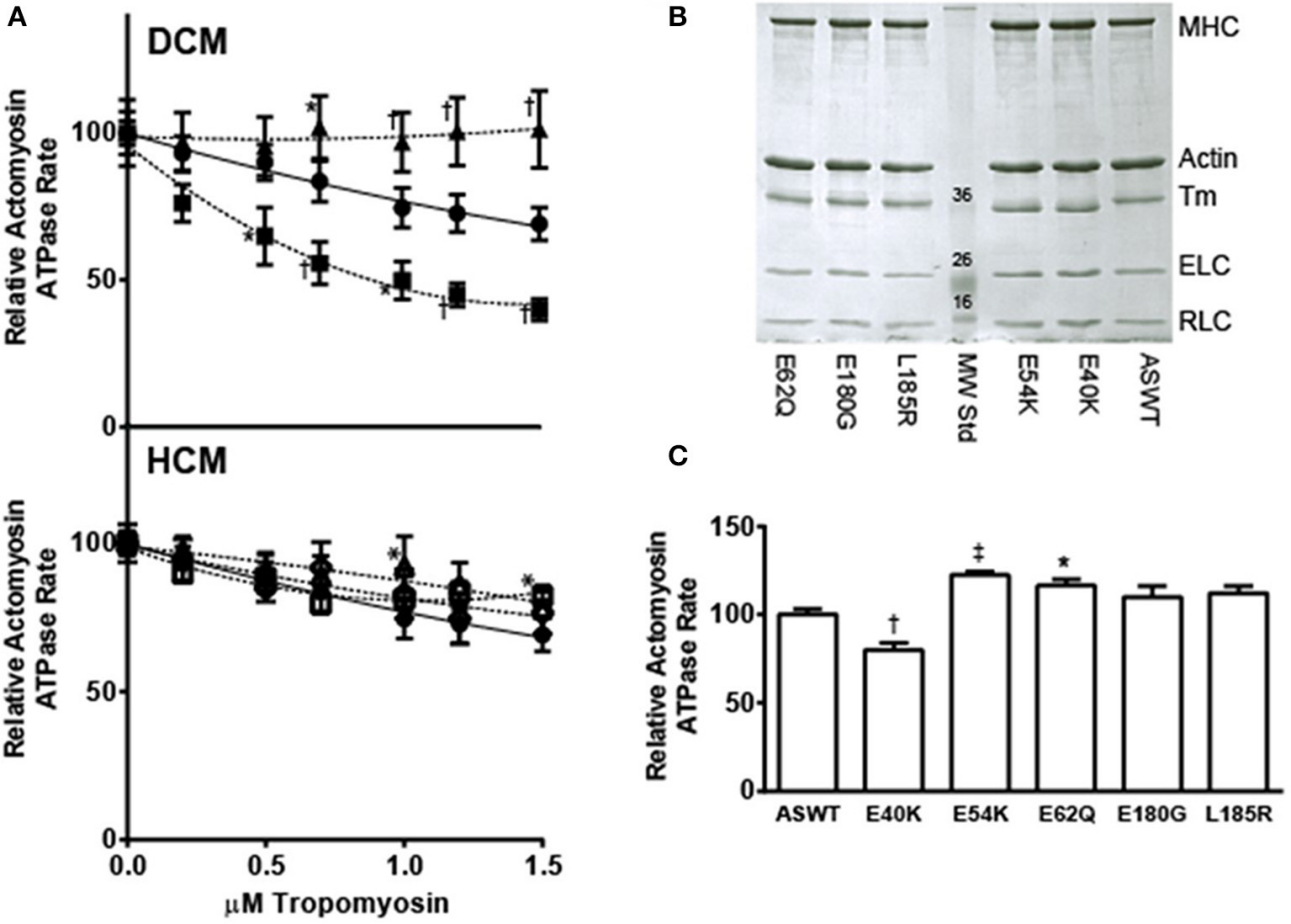
**Effects of DCM and HCM-associated mutations on Tm-dependent inhibition of actomyosin ATPase activity. (A)** The effects of Tm concentration dependent inhibition of actomyosin ATPase activity were compared between ASWT and DCM (upper panel) and HCM (lower panel) associated mutants. Tm was titrated from 0 to 1.5 μM into solutions of 0.6 μM myosin and 3.5 μM actin, in low Ca^2+^ (pCa 9.5) buffer, and the actomyosin ATPase activity measured. The results represent mean ± SE of activity rates normalized to the maximum ATPase activity at 0 μM Tm. All samples were measured in triplicate; *n* = 5. Samples graphed on DCM panel: ASWT (filled circle), E40K (filled square), E54K (filled triangle); on HCM panel: ASWT (filled circle), E62Q (open diamond), E180G (open square), L185R (open triangle). **(B)** Coomassie stained SDS-PAGE analysis of Tm titration study samples. Samples taken from actomyosin ATPase activity assay mixtures (0.6 μM myosin, 3.5 μM actin, 1 μM Tm) were run on a 12% SDS-PAGE gel, to confirm purity and proper ratios of proteins utilized in the Tm titration studies. **(C)** Comparison of relative ATPase rates in the presence of 1 μM Tm. All samples were measured in triplicate; *n* = 16. Statistical significance were determined by ANOVA followed by Dunnett's multiple comparisons test; ^*^p < 0.05, ^†^*p* < 0.01, ^‡^*p* < 0.001.

Increasing the concentration of Tm is known to result in diminished actomyosin ATPase activity, when the myosin concentration is held low and constant (Lehrer and Morris, 1982). To gain information on the possible effects of the mutations on Tm binding to actin and consequently, inhibition of actin-myosin interactions, actomyosin ATPase rates were measured at low [Ca^2+^] conditions with increasing amounts of Tm, in the absence of Tn (Figure 2A). Comparison of the Tm concentration dependent inhibition showed that E40K inhibited actomyosin ATPase activity better than ASWT, while E54K did not inhibit the actomyosin ATPase activity at all (Figure 2A). HCM associated mutants did not inhibit the actomyosin ATPase activity over ASWT, except the E62Q which increased the actomyosin ATPase activity (Figure 2A). Ratios of protein mixtures for ASWT and mutants were confirmed by Coomassie gel analysis (Figure 2B) and activity rates at 1 μM Tm compared for all mutants (Figure 2C). Consistent with trends seen in the Tm titration studies, and the baseline rate differences measured in the Tn titration studies, the rates with 1 μM Tm are significantly lower in E40K and significantly higher in E54K and E62Q mutants. The rates of E180G and L185R are increased but not significantly.

### Effects of mutations on Tm structure

**Figure 3:**
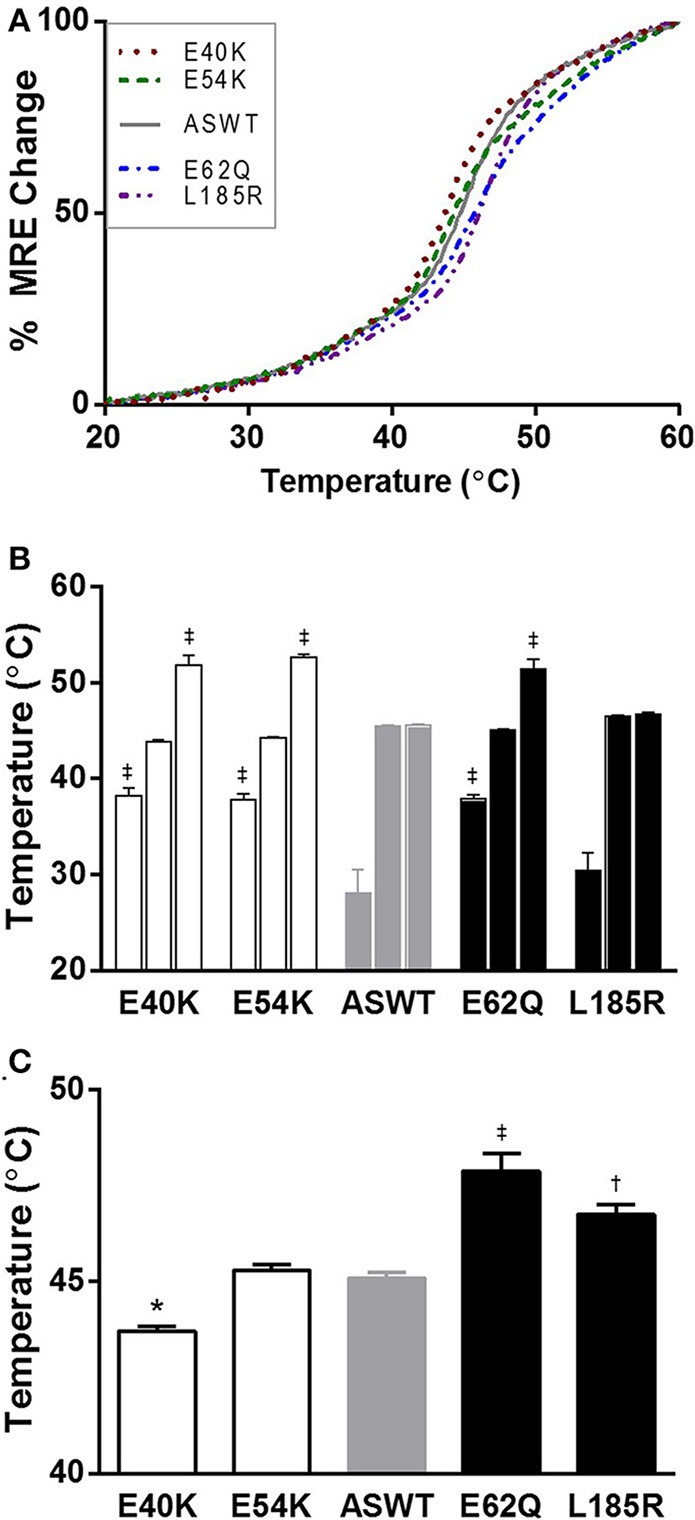
**Thermal denaturation spectra of ASWT and mutants. (A)** Purified Tms were denatured by increasing the temperature from 20 to 60°C, and the changes in ellipticity measured at each 0.2°C. Each spectra represents averaged mean residue ellipticity (MRE) of *n* = 9. **(B)** Thermal transition temperatures obtained from fitting the denaturation spectra to three transition models are shown as mean values ±s.e.m. **(C)** Half maximal denaturation temperatures for ASWT and mutants are shown as mean values ±s.e.m. Statistical significance were determined by ANOVA followed by Dunnett's multiple comparisons test; ^*^p < 0.05, ^†^*p* < 0.01, ^‡^*p* < 0.001.

The effects of the mutations investigated by CD spectra measurements showed that the single point mutations do not have significant effects on the mean residue ellipticity of Tm. Minor differences were seen, but are not significant (data not shown). Further investigation of the effects of the mutations on Tm structure by thermal denaturation showed interestingly, that the point mutations have differential effects on the melting transitions of Tm (Figure 3A). The individual denaturation curves were fitted to a three-transition model to identify the melting transition temperatures, the enthalpy change associated with unfolding and the fraction of the change in ellipticity due to each transition. The values are summarized in Table 1. In three of the cases, the results of the mutations caused the appearances of an additional transition, suggesting that the changes were local, and affecting a relatively small portion of the Tm molecule (Figure 3B, Table 1). To assess the effects of the mutations on Tm stability, T_M50_ values were calculated from the thermal denaturation curves of ASWT and mutants. The T_M50_ values show shifts temperatures suggestive of decreased stability for the DCM E40K mutant and increased stability for E62Q and L185R (Figure 3C). The stability for the E54K mutant did not change compared to ASWT (Figure 3C, Table 1).

**Table 1:** **Thermal transition temperatures of ASWT and mutants**.

**Mutant**	**T_M50_**	**T_M1_**	**T_M2_**	**T_M3_**	**ΔH_1_**	**ΔH_2_**	**ΔH_3_**	**ε_1_**	**ε_2_**	**ε_3_**	***n***
	**Degrees celsius**				**Kcal/Mol**						
ASWT	45.3 ± 0.3	28.1 ± 2.5	n/a	45.5 ± 0.1	−22 ± 8	n/a	−144 ± 29	0.38 ± 0.03	n/a	0.64 ± 0.04	9
E40K	44.0 ± 0.2^*^	38.2 ± 0.8^‡^	43.9 ± 0.2	51.8 ± 1.0^‡^	−38 ± 12	−146 ± 36	−128 ± 34	0.39 ± 0.08	0.41 ± 0.06	0.18 ± 0.05	9
E54K	45.3 ± 0.1	37.8 ± 0.6^‡^	44.3 ± 0.1	52.6 ± 0.3^‡^	−36 ± 10	−146 ± 23	−118 ± 22	0.39 ± 0.07	0.36 ± 0.06	0.24 ± 0.05	9
E62Q	47.9 ± 0.5^‡^	37.9 ± 0.4^‡^	45.1 ± 0.1	51.5 ± 1.0^‡^	−30 ± 7	−149 ± 64	−141 ± 48	0.35 ± 0.09	0.35 ± 0.11	0.29 ± 0.19	11
L185R	46.8 ± 0.3^*^	30.4 ± 1.9	n/a	46.5 ± 0.1	−24 ± 6	n/a	−155 ± 33	0.39 ± 0.01	n/a	0.61 ± 0.02	9

*Shown are the results of thermal denaturation spectra fitted to a two and three-transition model (see methods for description). The values represent mean ± SE of the values acquired from the fits. The melting temperature (T_M50_), transition temperatures (T_M1, 2, 3_), corresponding enthalpy values (ΔH) and extinction coefficients (ε) for each mutant are reported for the spectra measured the number of times shown (n). Statistical significance for temperatures were determined by ANOVA followed by Dunnett's multiple comparisons test*;

* *p < 0.05*,

‡ *p < 0.001*.

**Figure 4:**
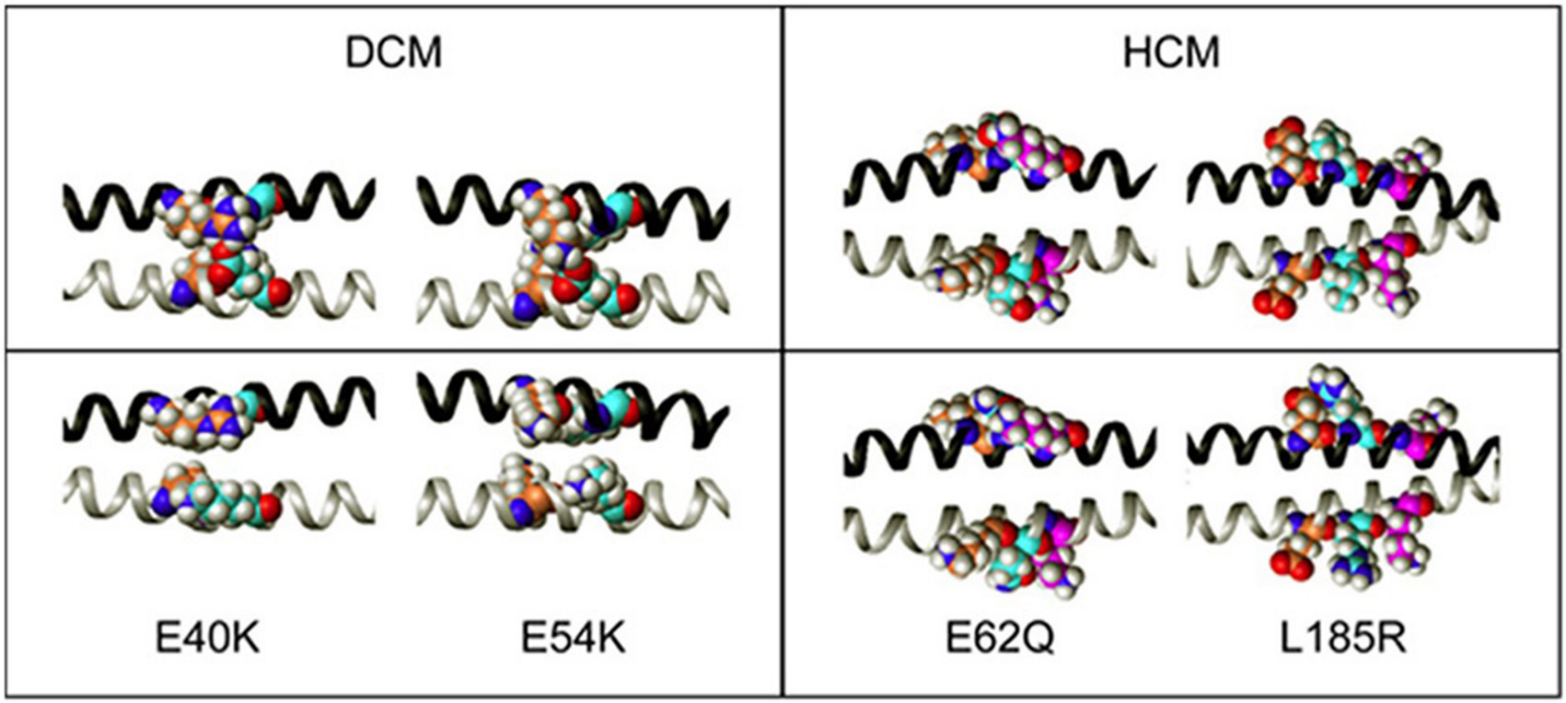
**The effects of DCM and HCM-associated mutations on the side-chain interactions of Tm dimers**. Side-chain interactions were modeled by Sybyl, and compared with the corresponding wildtype (upper half) for each mutation indicated. In all the panels the nitrogen atoms are blue and the oxygen atoms are red. The helical chairs are depicted as ribbons, with one chain of Tm in black and the other chain in gray.

Changes in Tm stability were explored through energy minimized models of the Tm mutations, which gives insight into the potential effects at the dimer interface (Figure 4). The heptad repeat position of the native protein residues and the expected interactions within the Tm molecule interface has been described in detail (Brown and Cohen, 2005). In the native protein, E40 in position *e* has a strong inter-chain interaction with R35 in position *g*, where the carboxyl group of E40 can interact with the amine side-chains of R35 (Brown et al., 2001, 2005). Destabilization of the coiled-coil by E40K is observed. In addition, E54 displays an intra-chain i to i+3 interactions with K51 and an inter-chain interaction with K49 in position *g*. Destabilization of the coiled-coil by E54K mutation is observed. Mutation of E62 to glutamine would not disrupt its interaction with K59 since the two could still form a strong hydrogen bond, and would allow interaction of the amide nitrogen with the carboxyl of D58, which could result in stabilization of the molecule. However, the carboxyl groups of E62 (carbon atoms in cyan) have intra-chain interactions with the amine groups of K65 (carbon atoms in magenta). When the E62 is mutated to Q, additional interactions occur; the carboxyl group of the amide side-chain of Q62 can still interact with the amine side-chain of K65 and the carboxyl groups of D58 (carbon atoms in coral) can now hydrogen bond to the amide hydrogens of the Q62 side chain. L185 is a hydrophobic residue that is exposed to the solvent and does not have any close inter- or intra-chain interactions. The side-chains of L185 and R185 do not interact with K189 (carbon atoms in magenta), but when L185 (carbon atoms in cyan) is mutated to R, it loses the entropic penalty of having a hydrophobic residue exposed to the solvent, and the basic side-chain can interact with the carboxyl groups of E181 (carbon atoms in coral), stabilizing the molecule.

## Discussion

The data presented here reports on the effects of various cardiomyopathy associated mutations on Tm structure and function. In the investigation of the effects of the mutations on Tn-dependent activation and inhibition of actomyosin ATPase activity, the results show that the effects of the HCM-associated mutations on the thin filament segregate well with disease, increasing maximal actomyosin ATPase rates (Figure 1C), as previously reported by myofibrillar ATPase assays (Chang et al., 2005). Structurally, the position of the HCM-associated mutation E62Q is an actin binding site residue (Phillips et al., 1986), and energy minimized structural models predicted an increase in Tm dimer stability, which is confirmed by the thermal denaturation study presented here (Figure 3C). Due to its position in the Tm coiled-coil, E62 does not have any inter-chain interactions in either the WT or mutant proteins. The position of the HCM-associated mutation L185R is also a consensus actin binding residue, and is in a region of Tm found to be critical for actin binding and regulation (Greenfield et al., 2002; Singh and Hitchcock-Degregori, 2006). Although there are local stability changes associated with E62Q which are not seen in L185R (Figure 3B), both mutations caused increases in overall stability of the Tm molecule (Figure 3C). Energy minimized model of the L185R mutant structure suggests the reason for stabilization of Tm consistent with increased T_M50_ of thermal denaturation (Figure 3C).

Consistent with our findings, several studies have reported that changes in Tm stability and consequently disinhibition of the myofilament at low [Ca^2+^] and increasing the number of active crossbridges is one of the mechanisms by which Tm mutations associated with HCM may induce diastolic dysfunction (Li et al., 2012; Ly and Lehrer, 2012). In terms of clinical manifestation, both HCM associated mutations were found in families which were greatly affected by the mutation as demonstrated by the segregation studies (Van Driest et al., 2002; Jongbloed et al., 2003). The L185R mutation has only been investigated by our group (Chang et al., 2005), and the recent identification of this mutation in several members of another family with HCM, including an 8-year-old girl who had sudden cardiac death (Makhoul et al., 2011), underscores the need for characterization of the molecular mechanisms affected by Tm mutations.

When the effects of the mutations are measured by myofibrillar ATPase assays, both DCM-associated mutations E40K and E54K had no effect on the inhibition of ATPase activity (Chang et al., 2005). Examination of the current Tn titration study shows that the baseline rates in the absence of Tn are different from ASWT for E40K and E54K (Figure 1A). While E40K has diminished activity in low [Ca^2+^], E54K has a slight increase (Figure 1B), in agreement with the results of the Tm titration study (Figure 2C). The highly increased inhibition of actomyosin activity by E40K and total lack of inhibition by E54K (Figure 2A), is suggestive of distinct mechanisms by which these two mutations cause DCM. Furthermore, the *in vitro* data for the E54K mutation correlate well with the *in vivo* phenotype reported in a transgenic mouse. The transgenic mice expressing the Tm E54K mutation demonstrated impaired systolic and diastolic functions (Rajan et al., 2007). The decreased Ca^2+^ sensitivity of the myofilament can be associated with the systolic dysfunction, while an inability of the heart to relax correlates with the solutions studies where the E54K does not inhibit the actomyosin ATPase (Figure 2A). This result does not agree with general findings reported for DCM-associated thin filament mutations, such as decreased Ca^2+^ sensitivity of contraction and decreased maximal force in reconstituted cardiac muscle fibers (Chang and Potter, 2005; Mirza et al., 2005; Willott et al., 2010), but is in agreement with previous studies by our group and others (Chang et al., 2005; Mirza et al., 2005, 2007). The possibility of the effects of E54K on actomyosin ATPase assays being an artifact or miscalculation is ruled out based on its agreement with other groups' results, and the fact that SDS-PAGE analysis shows that E54K Tm is present, and the amount of myosin is similar for all samples (Figure 2B).

Based on the sequence of Tm, and the crystal structures of Tm fragments (Brown et al., 2001, 2005), the following interactions may be affected by the mutations, and contributing to the differences in thermal stability. The mutation E40K occurs directly after a very unstable region of the Tm, which has alanines, lysines and serines in the *a* and *d* positions of the coiled coil interface, comprising a highly flexible region (Phillips, 1986). Like E40, E54 is an external glutamate which is postulated by Phillips to be a “consensus” actin binding residue (Phillips, 1986; Phillips et al., 1986). As postulated, both mutations E40K and E54K have been shown to reduce the affinity of Tm for actin, although at different thin filament states (On and Off states, respectively) (Mirza et al., 2007). Based on the actomyosin ATPase assay Tm titration studies, rather than substantially affecting the actin binding affinity (Mirza et al., 2007), the mutations may be affecting the position of Tm on actin itself, altering the blocked or open position of Tm on actin (Orzechowski et al., 2014). Indeed, the two mutations have been shown to affect the transition from weakly- to strongly-bound crossbridges when reconstituted into muscle fibers from which endogenous Tm was removed (Borovikov et al., 2009b, 2011). In the case of E40K, the position of Tm on actin may be blocking more of the myosin interaction sites than ASWT and thereby diminishing the baseline activity to a greater extent than ASWT. The decrease in maximal activity was not seen in the previously published myofibrillar ATPase assays (Chang et al., 2005), but based on the Tn and Tm ATPase inhibition studies presented here, obstruction of the myosin binding sites on actin likely exists, and this effect may be diminished in myofibrils due to the more intact nature of the system. In actomyosin ATPase assays, the effects of Tm alone on actomyosin interaction are masked by the presence of Tn, which confers Ca^2+^ sensitivity and increases the rate beyond the extent inhibited by Tm. In the case of E54K, Tn alone does not fully regulate the actomyosin ATPase activity, as evidenced by the increased activity over ASWT (Figure 1A), which is in agreement with another study, where increased cooperative unit size of actin-Tm and actin-Tm-Tn caused by E54K, was contrasted to a decrease caused by E40K, despite comparative phenotype (Olson et al., 2001) and decreases in Ca^2+^ sensitivity in more intact systems (Chang et al., 2005; Mirza et al., 2005). In a more intact system such as in myofibrils (Chang et al., 2005), where spatial organization of the thick and thin filaments are maintained, the mutation may be further diminishing the ATPase activity, possibly to the same extent of E40K, thus producing similar effects on contractility.

The results of the thermal denaturation studies suggest that a single point mutation does affect the global Tm structure, albeit not the total helical content, but the melting transitions of the Tm dimer itself. Characterization of these regions in Tm have shown that a denaturation curve of Tm, like those shown in Figure 3, is comprised of numerous overlapping melting transitions which correspond to flexible sequences that are essential for actin binding (Greenfield and Hitchcock-Degregori, 1995). The lack of significant differences in the secondary structure of Tm and the local destabilizations found in the denaturation curves suggest that the mutation induced alterations occur at the level of intra- and inter-coil interactions rather than at the level of the whole Tm dimer. However, when in complex with Tn, local instabilities in Tm are known to have long-range effects on the overlapping ends of Tm (Mamidi et al., 2013a,b). In conclusion, the findings reported here show that the structural alterations in Tm observed, may affect the position of Tm on actin, which in solution may alter the inhibitory or activating states of the thin filament. The results of the studies conducted suggest that in agreement with the wide range of clinical phenotypes, the mutational effects on structure and protein-protein interactions vary between and within disease categories.

### Conflict of interest statement

The authors declare that the research was conducted in the absence of any commercial or financial relationships that could be construed as a potential conflict of interest.

## Abbreviations

wild-type human α-striated tropomyosin with and N-terminal Ala-Ser dipeptide replacing the acetyl group (ASWT)
DCM: dilated cardiomyopathy
HCM: hypertrophic cardiomyopathy
Tm: tropomyosin
Tn: troponin.
